# Relationship of advanced glycation end-products in hypertension in diabetic patients: a systematic review

**DOI:** 10.1590/2175-8239-JBN-2022-0006en

**Published:** 2022-10-21

**Authors:** Joana Cortelete Fuhr, Maria Eduarda Kegler Ramos, Fabiana Piovesan, Luciana de Oliveira Renner, Luciano de Oliveira Siqueira

**Affiliations:** 1 Universidade de Passo Fundo, Instituto de Ciências Biológicas, Passo Fundo, RS, Brazil.; 2 Universidade de Passo Fundo, Faculdade de Medicina, Passo Fundo, RS, Brazil.

**Keywords:** Diabetes Complications, Diabetes Mellitus, Hypertension, Glycation End Products, Advanced, Systematic Review, Complicações do Diabetes, Diabetes Mellitus, Hipertensão, Produtos Finais de Glicação Avançada, Revisão Sistemática

## Abstract

Diabetes mellitus and arterial hypertension are among the five risk factors that increase mortality in the world. Both are chronic, non-communicable diseases (NCDs), that have a pathophysiological association. Advanced glycation end products (AGEs), produced by the lack of glycemic control in diabetic patients, interact with their AGE receptors (AGER) resulting in increased arterial stiffness, inflammation and endothelial changes - which increases the risk of developing hypertension and other complications. We ran a systematic review in Pubmed, SciELO, Cochrane Library and Web of Science databases using keywords and Boolean operators to optimize the search, with the objective of assessing the mechanism of non-enzymatic glycation of proteins present in patients with diabetes and its correlation with the onset of hypertension, exposing all the endothelial and cellular damage caused by AGEs. We found 719 papers, of which 99 were read in full, and 26 met the eligibility criteria and were included in the present review. AGEs should be considered one of the main cardiometabolic risk factors. Reducing the AGE-AGER interaction will result in cardiovascular protection and increased life expectancy.

## Resumo

Diabetes *mellitus* e hipertensão arterial estão entre os cinco fatores de risco que elevam a mortalidade no mundo. Ambas são doenças crônicas não transmissíveis (DCNT) que têm associação fisiopatológica. Os produtos finais de glicação avançada (AGEs), produzidos pela falta de controle glicêmico nos pacientes diabéticos, interagem com seus receptores para AGEs (RAGE) resultando no aumento da rigidez arterial e da inflamação e em alterações endoteliais, fatores que intensificam o risco do desenvolvimento da hipertensão e de demais complicações. Realizou-se uma revisão sistemática nas bases de dados Pubmed, SciELO, Cochrane Library e Web of Science utilizando descritores e operadores booleanos para otimizar a busca, com o objetivo de fornecer o mecanismo da glicação não enzimática de proteínas presente em pacientes com diabetes e sua correlação com o aparecimento da hipertensão, expondo todo o dano endotelial e celular ocasionado pelos AGEs. Foram encontrados 719 artigos, dos quais 99 foram lidos na íntegra, e 26 atenderam aos critérios de elegibilidade e foram incluídos na presente revisão. Os AGEs devem ser considerados um dos principais fatores de risco cardiometabólico. A redução da interação AGE-RAGE resultará na proteção cardiovascular e no aumento da expectativa de vida.

## Introduction

Diabetes mellitus (DM) and its associated complications represent a global problem in terms of human health and economy^1^. Diabetes affects 463 million people in the world today, and the projection for 2045, made by the International Diabetes Federation, is that it will reach more than 700 million people. This increase in prevalence and incidence is attributed to aging, sedentary lifestyle, smoking, urbanization and changes in the population’s diet^2^.

Hyperglycemia resulting from absolute or relative insulin deficiency can affect various tissues and organs of the body, causing chronic complications in multiple systems and organs, especially the cardiovascular system^1^. Micro and macrovascular complications can cause the endothelial dysfunction involved in the genesis of hypertension, commonly associated with diabetes. The combination of diabetes hyperglycemia and hypertension causes greater cardiovascular dysfunction, which becomes the main cause of morbidity and mortality from the disease^3^.

Studies show that there is a close relationship between diabetes and hypertension, and blood pressure elevation is twice as frequent in patients with diabetes compared to those without diabetes^4^. Common mechanisms, such as increased formation of advanced glycation end products (AGEs), activation of the receptor for advanced glycation end products (AGER), increased oxidative stress, chronic inflammation, endothelial dysfunction and activation of the renin-angiotensin system contribute to the close relationship between diabetes and hypertension^1^, ^4^.

AGEs are established as the main factors involved in the pathophysiology of diabetic vascular complications. Persistent hyperglycemia directly increases the formation of AGEs, resulting in inflammation, oxidative stress, vascular hyperpermeability, increased thrombogenicity and reduced vasorelaxation, which leads to homeostatic disturbance of the vasculature and consequent development of several late complications^5^, ^6^.

In this review, the association of diabetes and hypertension was argued by emphasizing the pathogenic role of AGEs in the development of hypertension in individuals with diabetes. We compiled the main articles and recently published experimental studies, aiming to demonstrate all the mechanisms present between AGEs and arterial hypertension (ah), and these mechanisms were not yet described in a single study as the present review showed. In addition to being a useful resource tool for researchers who wish to further investigate the role of AGEs in cardiovascular disorders correlated with DM.

We sought to expose evidence and biochemical mechanisms behind diabetes, hypertension and AGEs, instigating future projects for the development of pharmacological therapies to control AGEs in patients with diabetes aiming at cardiovascular protection and its complications.

## Methodology

### Study type

This is a systematic review on non-enzymatic glycation of proteins in the genesis of hypertension in patients with diabetes. This study aims to answer the guiding question formulated through the PICO strategy “What is the relationship of non-enzymatic glycation of proteins present in patients with diabetes and the development of hypertension?”. This systematic review is registered in the PROSPERO database as CRD42021246685.

### Eligibility criteria

We included papers published between 2016 and 2021 (last 5 years), in English and Portuguese, all related to the end-products of advanced protein glycation in diabetic patients with hypertension, without geographic or sample size restrictions.

There was no requirement for design type for the studies included, and all articles found from the search in the databases were evaluated using the pre-established keywords. Papers on type 2 diabetes mellitus were prioritized, excluding those that portrayed other types of diabetes.

Theses, dissertations, documents, letters and books were excluded from the review.

The papers were selected following an order: reading the title, reading the abstract and reading the full paper fo those in which the abstract met the inclusion criteria.

### Search Strategy

Searches were carried out in the Cochrane Library, PubMed/MEDLINE, SciELO and Web of Science databases by the authors [J.C.F] and [M.E.K], in July 2021, using the keywords selected in the Keywords in Health Sciences (KwHS): “Hypertension”, “Glycation End Products, Advanced“ and “Diabetes Mellitus”. Specific cross link associations were performed for each database, described in Table 1, and the Boolean operator [AND] was used to optimize the search.

**Table 1:** Search Strategies for the Selected databases

**MEDLINE / PubMed**	“Glycation End Products, Advanced” AND “hypertension”	“Glycation End Products, Advanced” AND “Diabetes Mellitus”	“Glycation End Products, Advanced” AND “Diabetes Mellitus” AND “hypertension”
**SciELO**	Glycation End Products, Advanced AND hypertension	Glycation End Products, Advanced AND Diabetes Mellitus	Glycation End Products, Advanced AND	Diabetes Mellitus AND hypertension
**Cochrane Library**	“Glycation End Products, Advanced” AND “hypertension”	“Glycation End Products, Advanced” AND “Diabetes Mellitus”	“Glycation End Products, Advanced” AND	“Diabetes Mellitus” AND “hypertension”
**Web of Science**	Glycation End Products, Advanced AND *hypertension	Glycation End Products, Advanced AND *Diabetes Mellitus	Glycation End Products, Advanced AND *Diabetes Mellitus AND *hypertension

Source: the author (2021).

### Study selection and data extraction

Paper selection was carried out by two independent authors [J.C.F] and [M.E.K]. The titles were transcribed into a worksheet, and duplicate articles were excluded. A thorough reading of titles and abstracts was carried out, so that those who met the aforementioned eligibility criteria made it to the final selection. Eligible papers were selected for full text reading and a new evaluation regarding the selection criteria.

Data extraction was performed by the authors together, compiling information, mechanisms and results from all articles included. The reviewer [L.O.S] did a thorough reading to rule out any discrepancies.

### Risk of bias

The articles included for writing the review went through a checklist to assess the quality and confidence of the results exposed by them. For this, we used the AMSTAR 2 checklist, proposed by Shea et al. The tool is a 16-item checklist for validation of randomized and non-randomized studies, being used to assess the methodological quality of systematic reviews or as a guide to carry out a systematic review^7^.

At the end of the analysis, the papers were classified as having high, moderate, low or critically low confidence in the results exposed by the study or review. To interpret the results, the tool proposed some critical items (2,4,7,9,11,13 and 15), that is, these items must be present in the paper and the rest of the items are considered non-critical, as they do not directly affect the quality of the study or review.

The paper had high confidence in the exposed results if it had none or one non-critical item marked; moderate confidence if more than one non-critical item is marked; low confidence if there is a critical item marked; and critically low confidence in the results if you have more than one critical item checked. As shown in Table 2.

**Table 2:** Papers included in the systematic review

Paper	Year	Study type	Study goal
(1)	2019	Literature review	Understand the relationship between oxidative stress and AGEs for the prevention and treatment of cardiovascular complications in patients with diabetes.
(2)	2017	Literature review	To review emerging evidence on the role of advanced glycation end-products (AGEs) in the diet as a cardiometabolic risk factor.
(3)	2017	Literature review	Understand the relationship between arterial stiffness and blood pressure.
(4)	2018	Literature review	Expose diabetes and hypertension as comorbidities and discuss the pathophysiological features of vascular complications associated with these conditions.
(5)	2018	Literature review	Associate AGEs to DM and coronary artery disease.
(6)	2020	Literature review	Expose the cellular and molecular basis of AGE-AGER axis signaling pathways in AGE-related diseases and discuss in detail the modes of action of newly discovered biomolecules and phytochemicals such as the Maillard reaction and the Signaling AGE-AGER inhibitors.
(8)	2019	Cross-sectional study with 282 participants without a prior diagnosis of diabetes	To investigate the correlation of the soluble receptor for advanced glycation end-products and endogenous secretory AGER (esAGER) with markers of cardiovascular disease in subjects with normal glucose tolerance and post-load glucose 1 h ≥ 155 mg/dL after an oral test of glucose tolerance.
(9)	2017	Cross-sectional study with 85 DM2 patients	To associate AGE intake with arterial stiffness, inflammatory profile and macronutrient composition in individuals with T2DM without evident cardiovascular disease.
(10)	2017	Literature review	Study in detail the effects caused by the activation of AGE receptors.
(11)	2020	Experimental study with 93 patients with DM and AH	To determine the serum levels of anti-AGEs antibodies in patients with DM and AH.
(12)	2016	Population experimental study with 1051 participants	To associate plasma AGE concentration with central and peripheral blood pressures and central to brachial blood pressure amplification in a Chinese population.
(13)	2018	Literature review	Explore the pathophysiological links between diabetes and hypertension.
(14)	2017	Literature review	To highlight the targets of AGEs in the heart and the mechanisms that lead to heart failure.
(15)	2016	Literature review	Gather the main studies that expose the main adverse effects of hyperglycemia.
(16)	2018	Experimental in vitro study with T-lymphocytes	Investigate the role of ICOS/ICOSL in the pathogenesis of T2DM.
(17)	2018	Experimental study using a subcohort and a case-control subgroup	To determine whether plasma levels of advanced glycation end-products and oxidation products (OP) predict the incidence of cardiovascular disease (CVD) in T2DM.
(18)	2020	Literature review	To expose the consequences of oxidative stress on skeletal muscle proteins in DM2.
(19)	2019	Literature review	To review the pathophysiological role of the AGE-AGER oxidative stress system and its therapeutic intervention in vascular damage in diabetes.
(20)	2019	Literature review	To highlight the consequences of the sustained increase in ROS production and inflammation that infuence the acceleration of atherosclerosis by diabetes.
(21)	2016	Experimental study with 122 patients with DM2	To evaluate the association of tissue AGE, as assessed by skin A F, with coronary artery calcification in Japanese subjects with type 2 diabetes.
(22)	2019	Case-control study in DM2 patients	To assess the association between the main types of advanced glycation end products in serum and selected serum/plasma markers of endothelial dysfunction in black patients with T2DM in a hospital.
(23)	2018	Literature review	Synthesize data from population studies on trends in diabetes complications.
(24)	2016	Experimental study with 862 participants	To evaluate the association of AGEs and arterial stiffening from the measurement by skin autofuorescence.
(25)	2019	Experimental in vitro study in macrophage	Use of methylglyoxal to investigate the infuence of glycation and AGEs on macrophages.
(26)	2016	Experimental study in mice	To investigate the effects of advanced glycation end products diet on diabetic vascular complications using diabetic mice.

Source: the author (2021).

This evaluation will not serve as an exclusion criterion for papers that have low confidence in the exposed results. It is just a measure of the quality of the papers available in the current literature.

## Results

We found 719 papers published in the last five years using the search strategies developed and researched in July 2021 in the four chosen electronic database. After analyzing the titles, following the eligibility criteria, we selected 102 papers. However, 3 papers were duplicated, leaving 99 papers for reading the abstract and the entire paper.

The main reasons for exclusion were papers with: 1) approaches to other late complications of diabetes, mainly nephropathy, neuropathy and Alzheimer’s; 2) other in vivo mechanisms linked to the onset of late complications of diabetes; 3) technologies for measuring AGEs in vivo; 4) pharmacological therapies to reduce AGEs. These papers did not provide the answer to the objective of the review, which is to evaluate the mechanism of non-enzymatic glycation of proteins for the onset of hypertension (late complication) in patients with diabetes.

**Figure 1:**
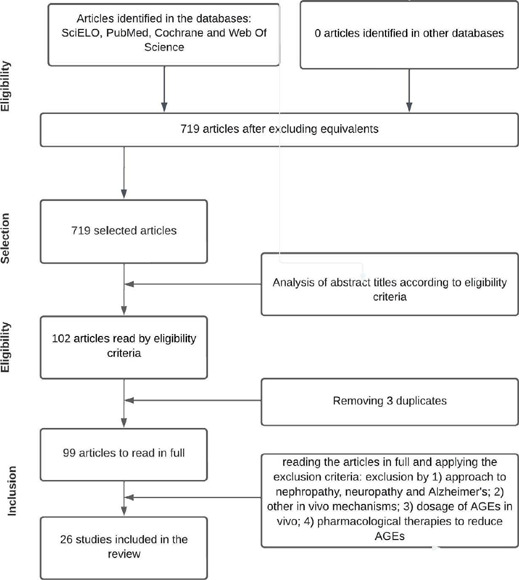
Flowchart showing the selection of papers for this review.

Finally, after a thorough assessment, 26 papers met the eligibility criteria for inclusion in this review, as summarized in Figure 1 and shown in Table 2 1, 2, 3, 4, 5, 6, 7, 8, 9, 10, 11, 12, 13, 14, 15, 16, 17, 18, 19, 20, 21, 22, 23, 24, 25, 26.

### Risk of bias

To analyze the risk of bias of the 26 articles included, we used the AMSTAR 27 checklist.

Items 7 and 8 are assigned to review papers, and experimental papers did not need to meet these criteria. Therefore, these items were excluded for the experimental papers included in this review.

Items 11, 12 and 15 are for papers that performed meta-analysis or quantitative synthesis. Therefore, for articles that did not perform meta-analysis or quantitative synthesis, these items were not assigned for the assessment of bias.

Item 13 was excluded, because the use of a specific method to assess bias in the included studies was not important in this review, provided that the study used some tool to reduce bias, an issue addressed in item 9.

The risk of bias analysis is shown in Table 3 below.

**Table 3:** Analysis of the risk of bias of the Table 3 studies included in the present study

Author/ Year	Item 1	Item 2	Item 3	Item 4	Item 5	Item 6	Item 7	Item 8	Item 9	Item 10	Item 11	Item 12	Item 13	Item 14	Item 15	Result
(1)	✓	✓	!	✓	!	!	!	✓	!	✓	-	-	✓	!	✓	**LOW**
(2)	✓	✕	✓	✓	✓	✓	!	✓	✕	✓	-	-	✓	-	✓	**C. LOW**
(3)	✓	✕	!	✓	!	!	✕	✕	✕	✓	-	-	✓	-	✓	**C. LOW**
(4)	✓	✓	✓	✓	✓	!	✓	✓	✓	✓	-	-	✓	✓	✓	**HIGH**
(5)	✓	✓	✓	✓	✓	✓	✓	✓	✓	✓	-	-	✓	✓	✓	**HIGH**
(6)	✓	✓	✓	✓	✓	✓	✓	✓	✓	✓	-	-	✓	!	✓	**V. LOW**
(8)	✓	✓	✓	✓	✓	!	-	-	✓	✓	✓	✓	✓	✓	✓	**HIGH**
(9)	✓	✓	✓	✓	✓	✓	-	-	✓	✓	✓	✓	✓	✓	✓	**HIGH**
(10)	✕	!	!	!	!	!	!	✓	✕	✓	-	-	✓	!	✓	**C. LOW**
(11)	✓	✓	✓	✓	✓	!	-	-	✓	✓	✓	✓	✓	✓	✓	**HIGH**
(12)	✓	✓	✓	✓	!	!	✕	✓	!	!	-	-	!	!	✓	**LOW**
(13)	✓	✓	✓	✓	!	!	!	!	✓	✓	-	-	✓	!	✓	**M. LOW**
(14)	✓	✓	✓	✓	✓	!	!	!	!	✓	-	-	✓	!	✓	**C. LOW**
(15)	✓	✓	✓	✓	✓	✓	!	✓	✓	✓	-	-	✓	✓	✓	**HIGH**
(16)	✓	✓	✓	✓	✓	!	-	-	✓	✓	✓	✓	✓	✓	✓	**HIGH**
(17)	✓	✓	✓	✓	✓	✓	-	-	✓	✓	✓	✓	✓	✓	✓	**HIGH**
(18)	✓	✓	✓	✓	✓	✓	✓	✓	✓	✓	-	-	✓	✓	✓	**HGIH**
(19)	✓	✕	✓	✓	!	!	✕	✓	!	✓	-	-	✓	!	✓	**C. LOW**
(20)	✓	✓	✕	✓	✓	✓	✕	✓	✕	✓	-	-	✓	-	✓	**C. LOW**
(21)	✓	✓	✓	✓	✓	!	-	-	✓	✓	✓	✓	✓	✓	✓	**HIGH**
(22)	✓	✓	✓	✓	✓	✓	✓	✓	✓	✓	-	-	-	✓	✓	**HIGH**
(22)	✓	✓	✓	✓	✓	✓	-	-	✓	✓	✓	✓	✓	✓	✓	**HIGH**
(23)	✓	✓	✓	✓	✓	✓	!	✓	✓	✓	-	-	✓	-	✓	**HIGH**
(24)	✓	✓	✓	✓	✓	✓	-	-	✓	✓	✓	✓	✓	✓	✓	**HIGH**
(25)	✓	✓	✓	✓	✓	✓	-	-	✓	✓	✓	✓	✓	✓	✓	**HIGH**
(26)	✓	✓	✓	✓	✓	!	-	-	✓	✓	✓	✓	✓	✓	✓	**HIGH**

Source: the author (2021).

1. Did the study questions and inclusion criteria include PICO components? 2. Did the review contain an explicit statement that the review methods were established and justified any significant deviations from the protocol? 3. Did the authors explain their selection of study designs? 4. Did the authors use a comprehensive literature search strategy? 5. Did the authors perform study selection in duplicate or was the selection of participants adjusted for interpersonal factors (gender/age/weight)? 6. Did the authors perform data extraction in duplicate? 7. Did the authors provide a list of excluded studies and justify the exclusions? 8. Did the authors describe the included studies in adequate detail? 9. Did the authors use a satisfactory technique to assess the risk of bias? 10. Did the authors report funding sources? 11. If a meta-analysis was performed, did the authors use appropriate methods for statistical combination of results? 12. If a meta-analysis was performed, did the authors use appropriate methods for statistical combination of results? 13. Did the review authors provide a satisfactory explanation for, and discussion of, any observed heterogeneity in the results? 14. If they performed a quantitative synthesis, did the authors perform an adequate investigation of publication bias and discuss its likely impact on the review results? 15. Did the authors report any potential sources of conflict of interest, including any funding they received to conduct the review?

**LEGEND:** ✓ **= Yes. ! = Not clear. ✕ = No.**

## Discussion

### Type ii diabetes mellitus and hypertension

Diabetes mellitus (DM) is a metabolic disorder characterized by persistent hyperglycemia, resulting from a deficiency in insulin production or action^27^, ^28^. Type 2 diabetes mellitus (DM2) has a multifactorial etiology, involving genetic and environmental components. Diet and physical inactivity contribute to the onset of obesity and stand out as the main risk factors for DM^22^.

DM and its associated complications represent a global problem for human health and the economy, which prevalence is increasing at an exponential rate worldwide^1^, ^23^. According to the International Diabetes Federation, DM is one of the fastest growing health challenges of the 21st century, because the number of adults with diabetes has more than tripled in the last 20 years. In 2019, there were 463 million adults with diabetes^28^.

It is expected that the number of adult patients with DM will continue to increase in the coming decades due to the increasingly frequent adoption of lifestyles associated with low energy expenditure and high caloric intake^2^. The number of patients with diabetes is estimated to increase to 700 million by 2045^28^.

Persistent hyperglycemia is associated with chronic micro and macrovascular complications that adversely affect the quality of life of patients with diabetes^1^, ^28^, and the main cause of morbidity and mortality in diabetes are cardiovascular diseases, which are potentiated by hypertension^3^.

Arterial hypertension (AH) is a highly prevalent multifactorial clinical condition, characterized by a sustained increase in blood pressure levels ≥ 140 and/or 90 mmHg. AH is often associated with metabolic disorders, functional and/or structural changes in target organs, being aggravated by the presence of other risk factors, such as dyslipidemia, abdominal obesity, glucose intolerance and DM^29^, ^30^.

In this context, DM and AH are chronic diseases easily found in the same individual. Consequently, both pathologies are closely linked as they have similar risk factors, such as dyslipidemia, sedentary lifestyle, obesity, insulin resistance and genetics^4^.

**Figure 2:**
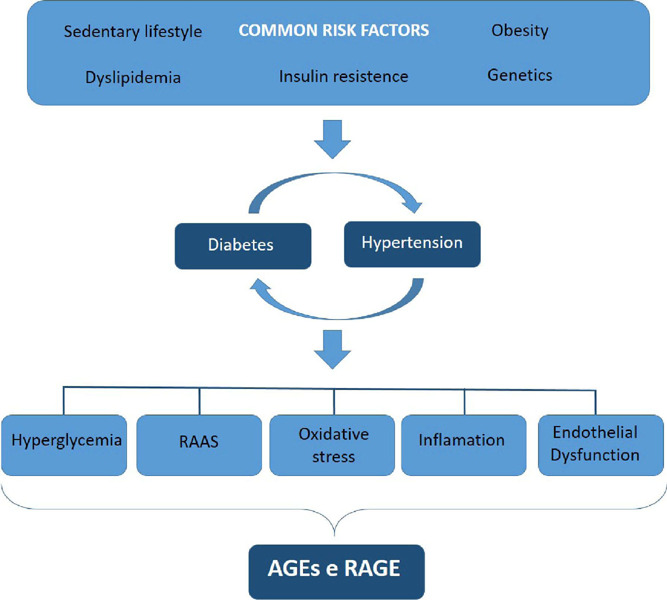
Interlink between DM and AH.

Source: adapted from Petrie et al. (2018).

The aforementioned risk factors activate mechanisms that cause late complications. The main biochemical mechanisms implicated in the genesis of hypertension in patients with diabetes include: increased formation of advanced glycation end products (AGEs), activation of the receptor for advanced glycation end products (AGER) (AGE-AGER axis), increased stress oxidative stress, chronic inflammation, endothelial dysfunction and activation of the renin-angiotensin system^1^, ^4^. The pathophysiology shared by both pathologies is summarized in Figure 2.

The main studies that prove these mechanisms in the development of hypertension in patients with diabetes are summarized in Table 4.

**Table 4:** Experimental Studies included showing the mechanisms associated with the development of cardiovascular complications in diabetic patients

Reference:	Model:	Result:	Methodology:
Hangai et al. (2016)^21^	Humans	**Increased AGEs:** Skin autofuorescence positively correlated with age, sex, duration of diabetes, pulse wave velocity, systolic blood pressure, serum creatinine, and cardiac calcium score.	One hundred and twenty-two Japanese subjects with type 2 diabetes were pooled to study the association of tissue AGE, assessed by skin autofuorescence (AF), with coronary artery calcification. They underwent multi-slice computed tomography to estimate of total coronary artery calcium scores (CACS) and examination with a skin autofuorescence scanner.
Zhang et al. (2018)^16^	In vitro	**Chronic inflammation:**	Hyperglycemia and AGES cause T cell-mediated inflammatory response and vascular endothelial dysfunction by upregulation of the ICOS/ICOSL protein	T-lymphocytes in peripheral human blood (CD3) and human endotelial cells from the umbilical vein (HUVECs) were treated with high glucose concentration and advanced glycation fnal products. ELISA and NOx production assays were used to detect the level of cytokines, cell feasibility and NOx production.
Mogale et al. (2019)^22^	Humans	**AGE increase** (carboximetyllisine) linked to higher likelihood of developing endothelial dysfunction.	Case-control study with type-II diabetic African patients, concluded that carboxymetyl-lisine (AGE) predisposes to endothelial dysfunction, through the analysis of serum/plasma markers of endothelial dysfunction.
Bezold et al. (2019)^25^	In vitro	**Chronic inflammation:**	Glycation caused an increase in macrophage cell AGE formation, increase in the expression of proinflammatory 1β interleukines (IL-1β) and IL-8, and affected the IL-10 and TNF-α expression, causing an increase in inflammation.	THP-1 human monocytic cells were cultivated, differentiated into macrophages by PMA 100 ng/mL and β-ME 50 µM. The macrophages were exposed to metylglioxal (MGO) to investigate the effect of cell glycation.
Koska et al. (2018)^17^	Humans	**Oxidative stress:**	Lower levels of MetSO (methionine sulfoxide) and higher levels of selected AGE are associated with increased incidence of cardiovascular disease (CVD) in type II diabetic patients.	Five specific AGEs and two oxidation products were measured at baseline in two intensive glucose-lowering studies: a subgroup from the Veterans Affairs Diabetes Trial (n=445) and a case-control subgroup from the Action to Control Cardiovascular Risk in Diabetes (n=271).
Di Pino et al. (2019)^8^	Humans	**AGE-esAGER:**	Individuals with 1h postload hyperglycemia have low plasma esAGER levels, increased pulse wave velocity and intima-media thickness.	Cross-sectional study with two hundred and eighty-two participants without a previous diagnosis of diabetes. We measured sAGER, esAGER and other markers of inflammation in subjects with 1h postprandial hyperglycemia and examined the association with early markers of cardiovascular damage.
Xing et al. (2016)^26^	Rodents	**AGE-AGER:**	Diet rich in AGEs increased 24hour urine protein levels, serum nitrogen, urea, creatinine, C-reactive protein, low-density lipoprotein, tumor necrosis factor α (TNF-α) and interleukin-6 ( IL-6) were also elevated. There was histological deterioration of the pancreas, heart and kidneys and caused structural changes to endothelial cells, mesangial cells and podocytes in the renal cortex.	Streptozocin-induced diabetic rodents (STZ) were fed a diet rich in AGEs. The characteristics of diabetes, indicators of renal and cardiovascular functions and the anatomopathology of the pancreas, heart and kidneys were evaluated.
Di Pino et al. (2017)^9^	Humans	**AGE-AGER and inflammation:**	Diet rich in AGE can lead to vascular dysfunction and inflammatory activation, contributing to the development of vascular complications in individuals with type 2 diabetes.	Arterial stiffness, carboxy-methyllysine, endogenous secretory receptor for AGEs (esRAGE), high-sensitivity C-reactive protein (hs-CRP), S100A12 and macronutrient intake were evaluated in 85 diabetic subjects.
Nikolov et al. (2020)^11^	Humans	**Anti-AGE antibodies sérum levels:**	Serum anti-AGE antibody levels in patients with type 2 diabetes mellitus and arterial hypertension were statistically significantly higher than in the control group, where determination of serum anti-AGE antibody levels can help clinicians make an early diagnosis and prognosis of the severity of late complications of diabetes in hypertensive patients.	ELISA was used to measure advanced glycation end-product antibody levels in serum from ninety-three patients with type 2 diabetes mellitus and high blood pressure.
Van Eupen et al. (2016)^24^	Humanos	**Skin autofluorescence correlated with aortic stiffening:**	The association between skin autofuorescence, pentosidine and femoral carotid pulse wave velocity were more pronounced in subjects with T2DM.	Eight hundred sixty-two participants (469 normal glucose; 140 altered glucose; 253 type 2 diabetes) were evaluated to determine the association of AGEs and arterial stiffening.

Source: the author (2021).

In healthy individuals, AGE levels are 3% lower than in individuals with diabetes. However, in patients with diabetes, this level can increase up to three times, resulting in the development of late complications^14^, ^31^.

Thus, AGEs are established as the main factors in the pathogenesis of vascular complications in diabetic patients. Hyperglycemia directly increases the formation of AGEs, which results in inflammation, oxidative stress, vascular hyperpermeability, increased thrombogenicity and reduced vasorelaxation, leading to homeostatic disturbance of the vasculature^5^, ^6^.

The study ran by Huang et al (2016) in Shanghai, China, on blood pressure levels correlated with AGEs of 1051 participants (388 men and 663 women) showed the connection between DM, AH and AGEs. In that study, plasma AGE concentration was positively associated with central systolic blood pressure, and in those diabetic and pre-diabetic individuals (90 participants) central systolic blood pressure was even more prominent^12^.

### Non-enzymatic glycation of proteinS in the genesis of hypertenSion in individuals with diabetes

#### Formation of age

Endogenous AGE formation occurs during physiological metabolism and normal aging by three independent pathways: the Maillard reaction, the polyol pathway, and during increased oxidative stress. During all three reactions, AGE synthesis leads to the formation of α-dicarbonyl compounds, such as glyoxal, methylglyoxal, 3-deoxyglucosone, glycolaldehyde and glyceraldehyde, which subsequently react with circulating proteins to form additional AGE molecules^10^, ^14^, ^30^.

**Figure 3:**
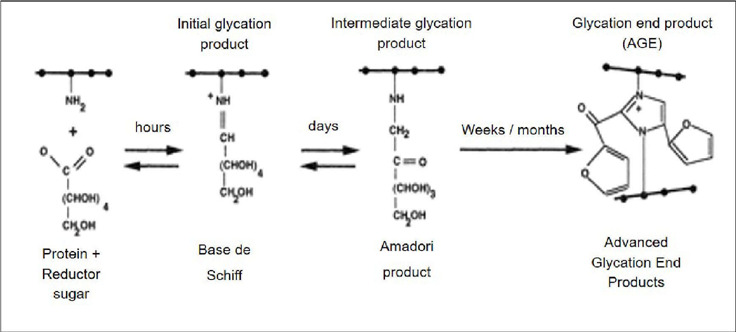
Representation of the advanced glycation end-product (AGE) formation.

The Maillard reaction, represented in Figure 3, presents the main source of AGE formation, in which the carbonyl part of a reducing sugar reacts with amino groups of proteins, lipids or nucleic acids to produce an unstable Schiff base that is later reorganized into a more stable ketosamine, the Amadori product. Amadori products can be transformed into α-dicarbonyls to yield AGE forms such as glucosepane, or oxidized to generate other AGE compounds such as carboxymethyllysine (CML) and pentosidine. Additional dehydration and oxidation reactions such as extensive crosslinking occur to generate more complex structures, crosslinked AGEs^30^.

#### Physiological effects of ages on the heart

The accumulation of AGEs in tissues occurs naturally during senescence, due to the decrease in protein turnover^30^. The extent of AGE formation in vivo is proportional to substrate availability as well as the rate of protein turnover. Long-lived proteins with significant lysine and arginine content (eg. collagen and elastin) are particularly susceptible to glycation. The normal physiological rate of AGE accumulation increases with advancing age, but is markedly increased in the presence of hyperglycemia, oxidative stress, and inflammation^5^.

AGEs provoke their cellular effects through three main changes: modification of extracellular proteins, modification of intracellular proteins, and cell-surface receptor-mediated signaling (RAGE)^14^, ^30^.

#### Modification of extracellular proteins

Modification of extracellular proteins by AGEs can alter the structure, function and properties of normal tissue, as well as provoke an inflammatory response. Collagen, elastin and laminin are key structural proteins of the basement membrane and connective tissue. Given their long half-life and amino acid composition, these molecules are highly susceptible to modification by AGEs^5^, ^19^.

AGEs alter the physiological properties of these extracellular matrix proteins through the formation of intermolecular bonds or crosslinking, affecting the mechanical properties of the target tissue which results in reduced elasticity, flexibility and promotes vascular and myocardial stiffness, contributing to impaired relaxation and diastolic dysfunction^14^.

Experimental studies carried out by Hangai (2016), Di Pino (2017) and Van Eupen (2016), cited in Table 4, prove the pathological role of AGEs in arterial calcification in patients with diabetes. Compiling the results of these studies, AGEs correlated with increased total coronary artery calcium score, increased pulse wave velocity, and altered systolic blood pressure, explaining the risk these patients have of developing cardiovascular disease.

Glycated collagen molecules are resistant to proteolytic digestion and form cross-links with other extracellular proteins, leading to decreased vessel wall flexibility and vascular stiffness. Glycation of elastin and laminin in the basement membrane has also been shown to impair endothelial cell adhesion and migration by disrupting cell attachment sites. These changes in cell-matrix interactions are associated with a stress-induced reduction in nitric oxide production by endothelial cells and impaired vasodilation^5^.

#### Modification of intracellular proteins

The intracellular accumulation of AGEs in the endoplasmic reticulum leads to cellular stress and can impair the normal processes of three-dimensional protein folding, generating inflammation or cellular apoptosis^30^.

Intracellular AGEs can bind to mitochondrial proteins of the respiratory chain involved in electron transport, decreasing ATP synthesis and increasing the production of superoxide and reactive oxygen species in cellular respiration^5^. In addition, glutathione peroxidase and glutathione reductase, enzymes of the antioxidant system, can be modified by AGEs, leading to a decrease in enzymatic activity, and thus favor a redox imbalance with a decrease in antioxidants^5^. Dicarbonyl is one of those AGEs that induces oxidative stress, suppresses these antioxidant enzymes and, therefore, causes cell death^18^.

Koska and collaborators (2018) proved the correlation between AGEs and increased oxidative stress through the study carried out with 716 participants. Five specific AGEs (methylglyoxal hydroimidazolone, carboxymethyl lysine, carboxyethyl lysine, 3-deoxyglucosone hydroimidazolone, and glyoxal hydroimidazolone) and two oxidative end-products (2-aminoadipic acid and methionine sulfoxide [MetSO]) were measured. Lower levels of MetSO (antioxidant) and higher levels of selected AGEs were found to be associated with increased incidence of cardiovascular disease (CVD) in patients with diabetes^17^.

AGEs are also capable of cross-linking the ryanodine receptor (RyR) domains of the sarcoplasmic reticulum Ca2+ATPase (SERCA) pump, leading to alterations in Ca2+ homeostasis, which results in a reduction in heart contractility^14^.

#### Linking age to its cell surface receptor for advanced end-product glylication (ager)

AGER belongs to the immunoglobulin superfamily of cell surface molecules, with binding affinity with various AGEs, as well as S100, amyloid and fibrillar protein aggregates, with pro-inflammatory molecules, among several other ligands. AGER is physiologically expressed in many types of cells, including macrophages, lymphocytes, fibroblasts, endothelial cells and cardiomyocytes^22^.

The interaction of AGE-AGER activates the nuclear factor (NF-κB), increases gene expression, the release of inflammatory cytokines and increases the production of reactive oxygen species (ROS), stimulating proliferative, fibrotic and thrombotic pathways that lead to vascular inflammation^3^, ^5^, linking AGER-mediated signaling to a series of pathogenic processes^14^.

The experimental studies carried out by Xing (2016) and Di Pino (2017), shown in Table 4, support the deleterious effect that the AGE/AGER interaction promotes. These studies demonstrate that binding increases the concentration of acute phase proteins such as C-reactive protein, tumor necrosis factor α (TNF-α) and interleukin-6 (IL-6), resulting in inflammatory activation. They also reported structural alterations of endothelial cells and deterioration of heart histology, characterizing vascular dysfunction.

In contrast, AGER exists in other isoforms, including soluble AGER (sAGER) that lacks the signal transducing peptide chain; endogenous secretory AGER (esAGER) and secreted human AGER (sechAGER). These AGER variants are present in the circulation, acting as scavengers of AGE molecules, being mainly involved in AGE clearance, that is, reduced levels of these variants may contribute to pathogenic results^30^.

Di Pino et al (2019) showed, in their cross-sectional study with 282 participants without a previous diagnosis of diabetes, that 1-hour postprandial hyperglycemia reduces plasma levels of esAGER, increases levels of AGEs and alters other markers of cardiovascular disease, concluding that the risk of cardiovascular disease increases before blood glucose reaches diabetic levels^8^.

### AGEs and hypertension

**Figure 4:**
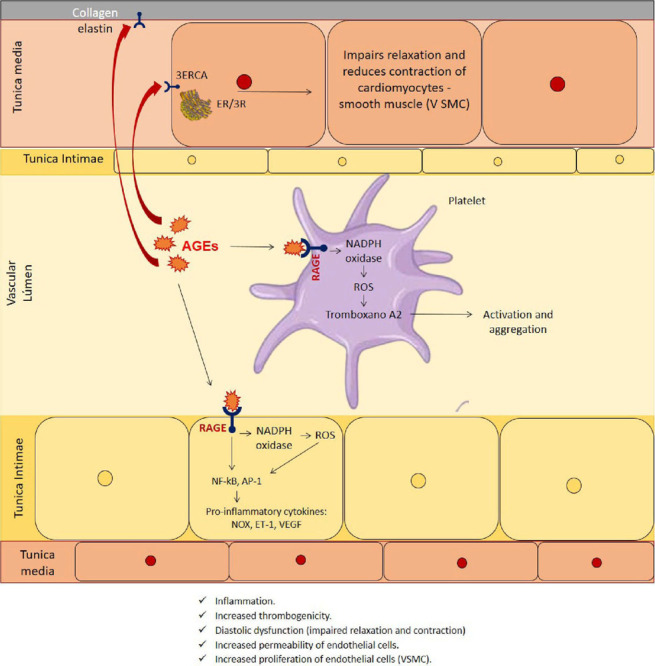
AGE-AGER integration effects on the coronary artery wall.

From the above, AGEs can induce hypertension in two ways: increasing arterial stiffness and promoting interaction of AGE with AGER on the cell surface, which results in changes in cell function and inflammation^2^. The effects on the coronary artery wall are exemplified in Figure 4.

### Changes in arterial rigidity

Arteries have two components: structural and dynamic. The structural component comprises the extracellular matrix, in which the causes of increased stiffness are the result of the modification in extracellular proteins, that is, the fragmentation and glycation of elastin and collagen increase and crosslinking^5^. These modifications alter the cell-matrix interactions and impair adhesion of endothelial cells. They can also reduce nitric oxide (NO) production and impair vasodilation, and these effects further contribute to decreased flexibility of vascular walls, subsequently leading to the diastolic dysfunction seen in patients with DM^19^, ^30^.

The dynamic component promotes arterial smooth muscle tone that depends on endothelial cell function (table 5). The endothelium releases vasoactive substances such as (NO) and endothelin-1 (ET-1). Normal endothelial function requires a balance between vasoconstrictors (ET-1) and vasodilators (NO). In insulin-resistance states, this balance is disrupted, as AGEs impair the NO production of endothelial cells by inhibiting the expression of endothelial NO synthesis, inhibiting vasodilator activity and increasing platelet aggregation and inflammation^3^, ^13^, ^30^.

**Table 5:** Dynamic and structural changes caused by AGEs onto the arterial wall

Structural changes (elastin and collagen)	Collagen reticulation (AGE-AGER collagen interaction) make it insolluble to hydrolithic enzymes, it is less succeptible to turnover, stiffer and increases its synthesis.	Elastin reticulation (AGE-AGER interaction elastin) reduces elasticity and reduces its synthesis.
Dynamic changes (endotelial cells)	AGEs reduce the NO vasodilator activity and bioavailability.	Impair or inactivate NO synthesis.	Reduce prostacyclin production.	Increase endothelin-1 expression.	Free radicals degrade elastin and collagen.

Source: the author (2021).

In addition, AGE-induced cross-linking was detected in intracellular proteins involved in Ca2+ homeostasis, such as the sarcoendoplasmic reticulum Ca2+ATPase pump and the ryanodine receptor (RyR). The crosslinking of the sarcoplasmic reticulum of the Ca2+ATPase pump impairs the Ca2+ content and affects cardiomyocyte relaxation, resulting in diastolic dysfunction. Crosslinking of RyR domains by AGEs also affects Ca2+ release and interrupts cardiomyocyte contraction^22^.

Neuroendocrine signaling can also compromise arterial stiffness. Angiotensin-II increases AGE formation and vice versa. Increased levels of angiotensin can then increase arterial stiffness through AGE or through the release of oxygen radicals through the interaction of AGE with AGER^3^.

### Endothelial dysfunction and inflammation

AGEs bind to AGERs activating several intracellular pathways that increase oxidative stress and proinflammatory molecules^2^, ^8^, ^9^, ^16^, ^17^, ^23^, ^26^, ^27^.

AGER activation triggers several signaling cascades: mitogen-activated protein kinases (MAPKs), nicotinamide adenine dinucleotide phosphate oxidase (NADPH) and a complex of enzymes that increase the production of reactive oxygen species (ROS). These signaling cascades trigger the activation and translocation of nuclear factor (NF-κB) from the cytoplasm to the nucleus. Thereafter, NF-κB triggers gene transcription for various pro-inflammatory cytokines, such as IL-1α, IL-6, and TNF-α, growth factors, and adhesion molecules, such as intercellular-1 (ICAM-1), vascular cell adhesion molecule-1 (VCAM-1), ET-1, tissue factor, vascular endothelial growth factor (VEGF). These cytokines and adhesion molecules have roles in both inflammation and endothelial dysfunction. RAGE transcription is also regulated by NF-κB. Therefore, the AGE-RAGE interaction promotes the maintenance and amplification of the signal with a sustained induction of the inflammatory response, the prothrombotic activity and the expression of adhesion molecules^2^, ^20^.

Furthermore, the accumulation of AGEs in the vascular lumen affects the activity and aggregation of platelets through AGE-AGER binding, and after AGE binds to AGER, NADPH hyperactivity is observed, leading to the generation of ROS, which is associated with increased cyclooxygenase activity and generation of thromboxane A2 (TXB) in platelets, contributing to thrombus formation^30^.

Briefly, endothelial cells under hyperglycemic conditions induce oxidative stress, activating NF-κB in AGER, where AGEs will bind, leading to upregulation of MAPK pathways. The AGE-AGER binding leads to the activation of NADPH and nitric oxide synthase (NOS), perpetuating a cycle of production of reactive oxygen species (ROS), pro-inflammatory cytokines and vascular adhesion molecules, thus decreasing endothelial homeostasis and cell damage^20^, ^30^.

Our study presents, firstly, the low quality of the papers available in the literature. As seen in Table 2, of the 26 papers included in this review, 6 papers (23.1%) had critically low confidence in the exposed results; 2 papers (7.7%) had low confidence in the exposed results; 2 papers (7.7%) had moderately low confidence in the exposed results; and 16 papers (61.5%) showed high confidence in the results, according to the criterion proposed by Shea (2017). Raising a criticism about the reliability of the results that are being exposed by the literature.

We emphasize the importance of updated scientific publications to support and clarify the association between the products of non-enzymatic glycation of proteins present in DM with the development and aggravation of its complications, especially AH, to prevent and delay them.

Evidence suggests that the determination of serum AGE may be useful as a biomarker for the presence and severity of cardiovascular disease, being even more valid for those patients with problems that increase the amount of circulating AGEs, as is the case of patients with diabetes^21^. However, further studies are needed to assess the usefulness of circulating and tissue levels of AGE in identifying patients at risk for cardiovascular disease.

Through the evidence and mechanisms demonstrated, it is urged that new studies be developed for the development of therapeutic interventions aimed at controlling AGEs in patients with diabetes, for cardiovascular protection and seeking a new way to prevent and care against the development of late complications from diabetes, to increase the quality and life expectancy of these patients.

## Conclusion

Prolonged periods of hyperglycemia increase the endogenous formation of AGEs. The AGE-AGER axis is involved in increased arterial stiffness, inflammation and endothelial changes - factors that increase the risk of developing hypertension in individuals with diabetes. Consequently, AGEs should be considered one of the main cardiometabolic risk factors, so control of risk factors common to pathologies and strategies to promote vascular health are essential for reducing microvascular and macrovascular complications of diabetes.
